# Reinterpretation of anthocyanins biosynthesis in developing black rice seeds through gene expression analysis

**DOI:** 10.1371/journal.pone.0286539

**Published:** 2023-06-02

**Authors:** Choonseok Lee, Yang-Seok Lee, Ha-Cheol Hong, Woo-Jong Hong, Hee-Jong Koh, Ki-Hong Jung

**Affiliations:** 1 Department of Genetics and Biotechnology and Crop Biotech Institute, Kyung Hee University, Yongin, Gyeonggi-do, Republic of Korea; 2 School of Life Sciences, University of Warwick, Coventry, United Kingdom; 3 National Institute of Crop Science, Wanju, Jeollabuk-do, Republic of Korea; 4 Graduate School of Green-Bio Science and Crop Biotech Institute, Kyung Hee University, Yongin, Gyeonggi-do, Republic of Korea; 5 Department of Agriculture, Forestry and Bioresources, Research Institute for Agriculture and Life Sciences, and Plant Genomics and Breeding Institute, Seoul National University, Seoul, Republic of Korea; ICAR - National Research Center on Plant Biotechnology, INDIA

## Abstract

The biosynthesis of anthocyanins is still questionable in regulating the quantities of anthocyanins biosynthesized in rice seeds and the expression levels of transcription factors and the structural genes involved in the biosynthetic pathway of anthocyanins. We herein investigated the relationship between the accumulated anthocyanin contents and the expression levels of genes related to the biosynthesis of anthocyanins in rice seeds. Liquid chromatography/mass spectrometry-mass spectrometry analysis of cyanidin 3-glucoside (C3G) in rice seeds showed no accumulation of C3G in white and red rice cultivars, and the differential accumulation of C3G among black rice cultivars. RNA-seq analysis in rice seeds, including white, red, and black rice cultivars, at twenty days after heading (DAH) further exhibited that the genes involved in the biosynthesis of anthocyanins were differentially upregulated in developing seeds of black rice. We further verified these RNA-seq results through gene expression analysis by a quantitative real-time polymerase chain reaction in developing seeds of white, red, and black rice cultivars at 20 DAH. Of these genes related to the biosynthesis of anthocyanins, *bHLH*s, *MYB*s, and *WD40*, which are regulators, and the structural genes, including *chalcone synthase* (*CHS*), *flavanone 3-hydroxylase* (*F3H*), *flavonoid 3´-hydroxylase* (*F3*´*H*), *dihydroflavonol 4-reductase* (*DFR*), and *anthocyanidin synthase* (*ANS*), were differentially upregulated in black rice seeds. The correlation analysis revealed that the quantities of C3G biosynthesized in black rice seeds were positively correlated to the expression levels of *bHLH*s, *MYB*s and *WD40*, *CHS*, *F3H*, *F3*´*H*, *DFR*, and *ANS*. In addition, we present *bHLH2* (*LOC_Os04g47040*) and *MYB*s (*LOC_Os01g49160*, *LOC_Os01g74410*, and *LOC_Os03g29614*) as new putative transcription factor genes for the biosynthesis of anthocyanins in black rice seeds. It is expected that this study will help to improve the understanding of the molecular levels involved in the biosynthesis of anthocyanins in black rice seeds.

## Introduction

Anthocyanins, a class of flavonoids [1], have been identified in various plant species with their specific anthocyanin(s), i.e., malvidin 3-galactoside in *Primula polyanthus* [2], peonidin 3-glucoside in *Oxycoccus macrocarpus* [3], pelargonidin 3-glucoside in *Fragaria chiloensis* [4], cyanidin 3-glucoside in *Rumex crispus* [5] and *Spirodela intermedia* [6], glucosides of cyanidin, delphinidin, malvidin and petunidin in *Medicago sativa* [7], cyanidin 3-glucoside, peonidin 3-glucoside, cyanidin 3-rutinoside and cyanidin 3-galactoside in *Oryza sativa* [8–10]. It has been known that anthocyanins are induced in plants by biotic [11] or abiotic [5, 12, 13] stress and have an antioxidative activity [14, 15].

In the analysis of anthocyanins in various organ/tissue samples, including leaf blade, leaf sheath, collar, internode, auricle, ligule, hull, apiculus, and pericarp, of Purpleputtu, a black rice cultivar, Reddy et al. reported cyanidin as a major anthocyanidin and peonidin, a 3´-methoxy cyanidin derivative as a minor anthocyanidin [8]. Yoshimura et al. exhibited that black rice seeds contain cyanidin 3-glucoside (C3G), a major anthocyanin, and peonidin 3-glucoside (P3G), a minor anthocyanin. They accumulate in the outer pericarp and seed coat layer of black rice seeds. They further reported various glucosides of cyanidin, peonidin (3´-methoxy cyanidin), petunidin (3´-hydroxy-5´-methoxy cyanidin), and malvidin (3´,5´-dimethoxy cyanidin) [10].

**Fig 1:**
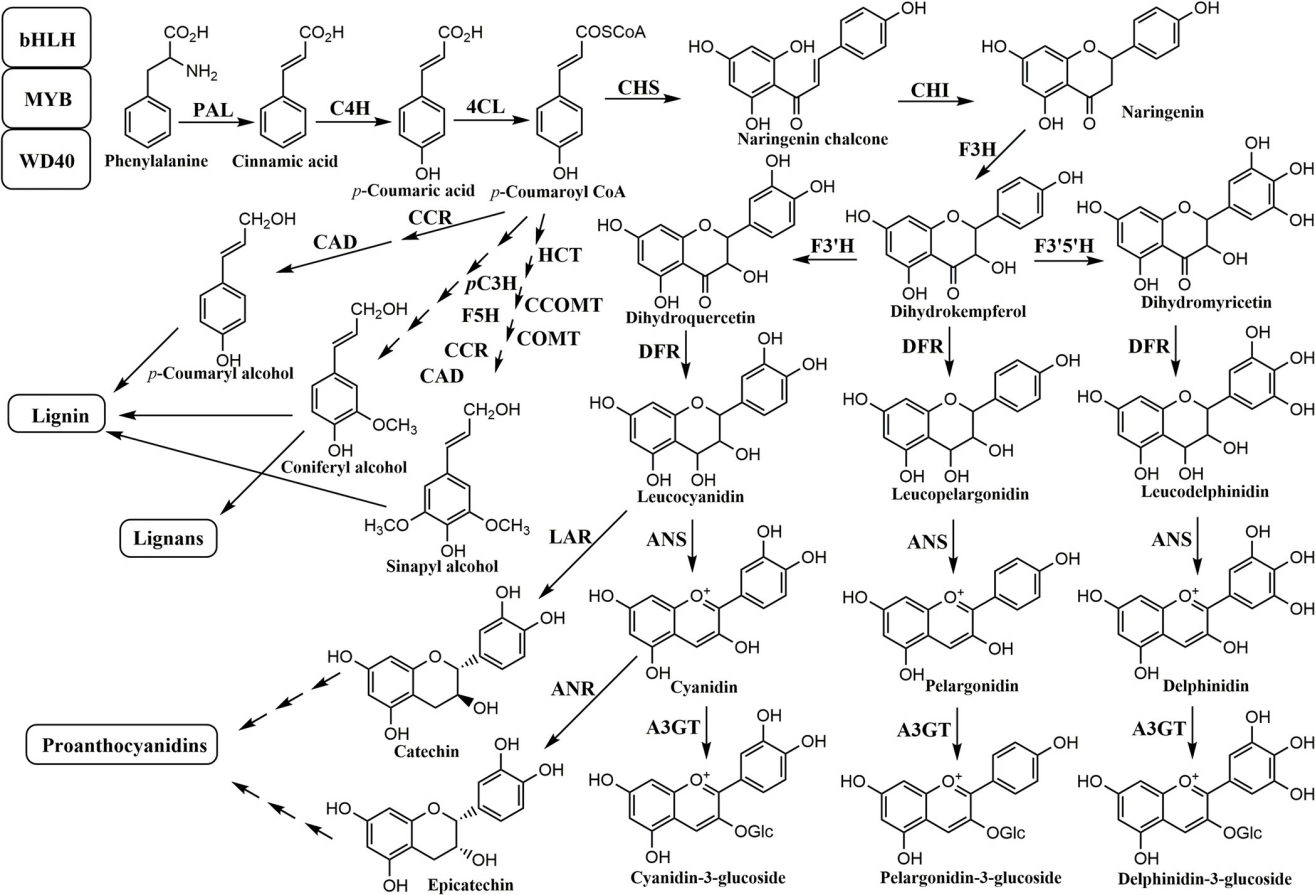
Schematic representation of the biosynthetic pathway of anthocyanins in plants. bHLH: basic helix-loop-helix protein; MYB: myb protein; WD40: tryptophan-aspartic acid repeat protein; PAL: phenylalanine ammonia-lyase; C4H: cinnamate 4-hydroxylase; 4CL: 4-coumarate: CoA ligase; CCR: cinnamoyl-CoA reductase; CAD: cinnamyl alcohol dehydrogenase; HCT: hydroxycinnamoyl-CoA shikimate/quinate hydroxycinnamoyltransferase; *p*C3H: *p*-coumarate 3-hydroxylase; CCOMT: caffeoyl-CoA *O*-methyltransferase; COMT: caffeate *O*-methyltransferase; F5H: ferulate 5-hydroxylase; CHS: chalcone synthase; CHI: chalcone isomerase; F3H: flavanone 3-hydroxylase; DFR: dihydroflavonol 4-reductase; F3´H: flavonoid 3´-hydroxylase; F3´5´H: flavonoid 3´,5´-hydroxylase; ANS: anthocyanidin synthase; LAR: leucoanthocyanidin reductase; ANR: anthocyanidin reductase; A3GT: UDP-glucose: anthocyanidin 3-*O*-glucosyltransferase.

It was first known in *Zea mays* that the biosynthesis of anthocyanins is regulated by two transcription factors, including *C1* [16, 17], a myb gene, and *R* [18], a basic helix-loop-helix (bHLH) gene [19]. Orthologous genes to these two genes were also reported in rice [20, 21]. The biosynthetic pathway of anthocyanins has been well elucidated in plants, as presented in Fig 1. The first step of the biosynthesis of anthocyanins is the conversion of *p*-coumaroyl CoA, formed from phenylalanine via stepwise reactions by phenylalanine ammonia-lyase (PAL) [22], cinnamate 4-hydroxylase (C4H) [23, 24], and 4-coumarate: CoA ligase (4CL) [25], respectively, and malonyl CoA into naringenin chalcone by chalcone synthase (CHS) [26, 27]. Naringenin chalcone is finally converted into several kinds of anthocyanins via several reactions by chalcone isomerase (CHI) [28–30], flavanone 3-hydroxylase (F3H) [31, 32], dihydroflavonol 4-reductase (DFR) [33–35], flavonoid 3´-hydroxylase (F3´H) [36, 37], flavonoid 3´,5´-hydroxylase (F3´5´H) [38], anthocyanidin synthase (ANS) [39–41], and UDP-glucose: anthocyanidin 3-*O*-glucosyltransferase (A3GT) [40, 42, 43], respectively (Fig 1).

Compared to developing seeds of white rice with no anthocyanin accumulation, *CHS*, *F3H*, *DFR*, and *ANS* were upregulated in developing black rice seeds [44] and *Kala4* (*LOC_Os04g47059*), an orthologous *bHLH* gene of maize *R* gene, was also upregulated in black rice seeds [45]. Among black rice cultivars, there were significant differences in the quantities of anthocyanins, C3G and P3G, in mature rice seeds [46]. However, in developing seeds of black rice, there needs to be more information on the relationship between the quantities of anthocyanins biosynthesized and the expression level of genes related to the biosynthetic pathway of anthocyanins. In this study, we investigated how the biosynthesis of anthocyanins is related to the expression of genes involved in the biosynthetic pathway of anthocyanins in developing seeds of black rice.

## Materials and methods

### Growth of rice cultivars used in this study

We transplanted and cultivated three replicates of seedlings of Dongjin (white rice), Geonganghongmi (red rice), Jeokjinju (red rice), Boseokheukchal (black rice), Heukjinju (black rice), Heukjinmi (black rice), and Heukseol (black rice) in the experimental paddy field of the National Institute of Crop Science (NICS), Republic of Korea, by a completely randomized design using the standard rice cultivation method of the NICS. We sampled developing seeds from one panicle per plant and four plants per replicate at twenty days after heading (DAH), and harvested mature seeds at around sixty DAH.

### Quantification of cyanidin 3-glucoside by liquid chromatography/ mass spectrometry-mass spectrometry

We quantified cyanidin 3-glucoside (C3G) content in the flour of hulled rice with three replicates of Dongjin, Geonganghongmi, Jeokjinju, Boseokheukchal, Heukjinju, Heukjinmi, and Heukseol using the methods developed in the Center for University-Wide Research Facilities at the Jeonbuk National University with liquid chromatography/mass spectrometry-mass spectrometry [LC/MS-MS, Xevo TQ-S triple quadrupole mass spectrometer (Waters Corporation, Milford, USA) coupled with ACQUITY Ulta-Performance Liquid chromatography system (Waters Corporation, Milford, MA, USA)] [47].

We purchased the standard material, cyanidin 3-glucoside, from Sigma-Aldrich (Saint Louis, MO, USA).

### Total RNA extraction and RNA-seq

We extracted total RNA from the frozen and milled samples with three replicates of developing seeds of Dongjin, Geonganghongmi, Jeokjinju, Boseokheukchal, Heukjinju, Heukjinmi, and Heukseol at 20 DAH using the RNeasy Plant Mini Kit (QIAGEN, Hilden, Germany) with the manufacturer’s instructions. Of these total RNA samples, we sent each one replicate of the total RNA samples of Dongjin (white rice), Jeokjinju (red rice), and Heukseol (black rice) to Macrogen, Inc (Seoul, Republic of Korea) for RNA-seq using the Illumina technology by paired-end type sequencing with 101-bp read length.

We processed the raw data of RNA-seq by the methods described by Lee et al. [48]. Briefly, the raw data were quality trimmed using the Cutadapt software with parameters: -a AGATCGGAAGAGC–A AGATCGGAAGAGC–q 30 –m 20 [49], and the trimmed data were mapped to a reference rice genome, MSU7 (http://rice.uga.edu/) using the HISAT2 software [50] with default parameter (S1 Table). Read counts data were calculated with the featureCounts software [51] (S2 Table). We finally obtained normalized read counts data from the processed raw data of RNA-seq by division of the read counts of all genes with those of the *OsUBI1* gene (*LOC_Os03g13170*) (S2 Table). The raw data of RNA-seq are available at https://www.ebi.ac.uk/arrayexpress/experiments/E-MTAB-9993.

### Gene expression analysis by quantitative real time-polymerase chain reaction

As described by Lee et al. [48], we synthesized cDNA using the iScript^TM^ cDNA Synthesis Kit (Bio-Rad, Hercules, CA, USA) from 1 μg of each total RNA taken in total RNA samples with three replicates of Dongjin, Geonganghongmi, Jeokjinju, Boseokheukchal, Heukjinju, Heukjinmi, and Heukseol. We carried out quantitative real time-polymerase chain reaction (qRT-PCR) with cDNA and the primer sets listed in the S3 Table in the CFX96^TM^ Real-Time Detection System (Bio-Rad, Hercules, CA, USA) using iQ SYBR Green Supermix (Bio-Rad, Hercules, CA, USA). We used the *OsUBI1* gene, *LOC_Os03g13170*, as a reference gene (S3 Table) [52–54]. We used the Pfaffl [55] method to determine the relative expression of genes described in the S3 Table.

### Generation of heatmap

We generated all heatmaps with log_2_(FPKM+1) values using the pheatmp package in R version 1.2.5033 (Kolde, 2018; RRID:SCR_016418). We obtained FPKM values for various rice organs of Nipponbare (white rice) from the Rice Genome Annotation Project (http://rice.uga.edu/).

### Statistical analysis

We performed an analysis of variance (ANOVA) and Duncan’s Multiple Range Test (DMRT) using SAS9.4 TS Level 1 M5 (Ver.1.0.19041; SAS Institute Inc., Cary, NC, United States). We used the package corrplot in R version 1.2.5033 to conduct correlation analysis [56].

## Results

### Quantification of cyanidin 3-glucoside in seeds of black rice cultivars through LC/MS-MS

The National Institute of Crop Science (NICS) has developed and released fourteen black rice cultivars (S3 Table). Of these fourteen black rice cultivars, we selected four black rice cultivars, including Boseokheukchal, Heukjinju, Heukjinmi, and Heukseol, with consideration of quantities of C3G in hulled seeds and original parent(s) used in the breeding program for improvement of black rice traits. Interestingly, the genome of Heukjinmi partially retains the genomic content of Hongjinju, a red rice cultivar, as one of the parents in its breeding pedigree (S4 Table, http://www.nics.go.kr/api/breed.do?m=100000128&homepageSeCode=nics). We also selected one white rice cultivar, Dongjin, and two red rice cultivars, Geonganghongmi and Jeokjinju, as a control to compare the metabolic differences with black rice cultivars from the database for cultivars developed by the NICS (http://www.nics.go.kr/api/breed.do?m=100000128&homepageSeCode=nics).

**Fig 2:**
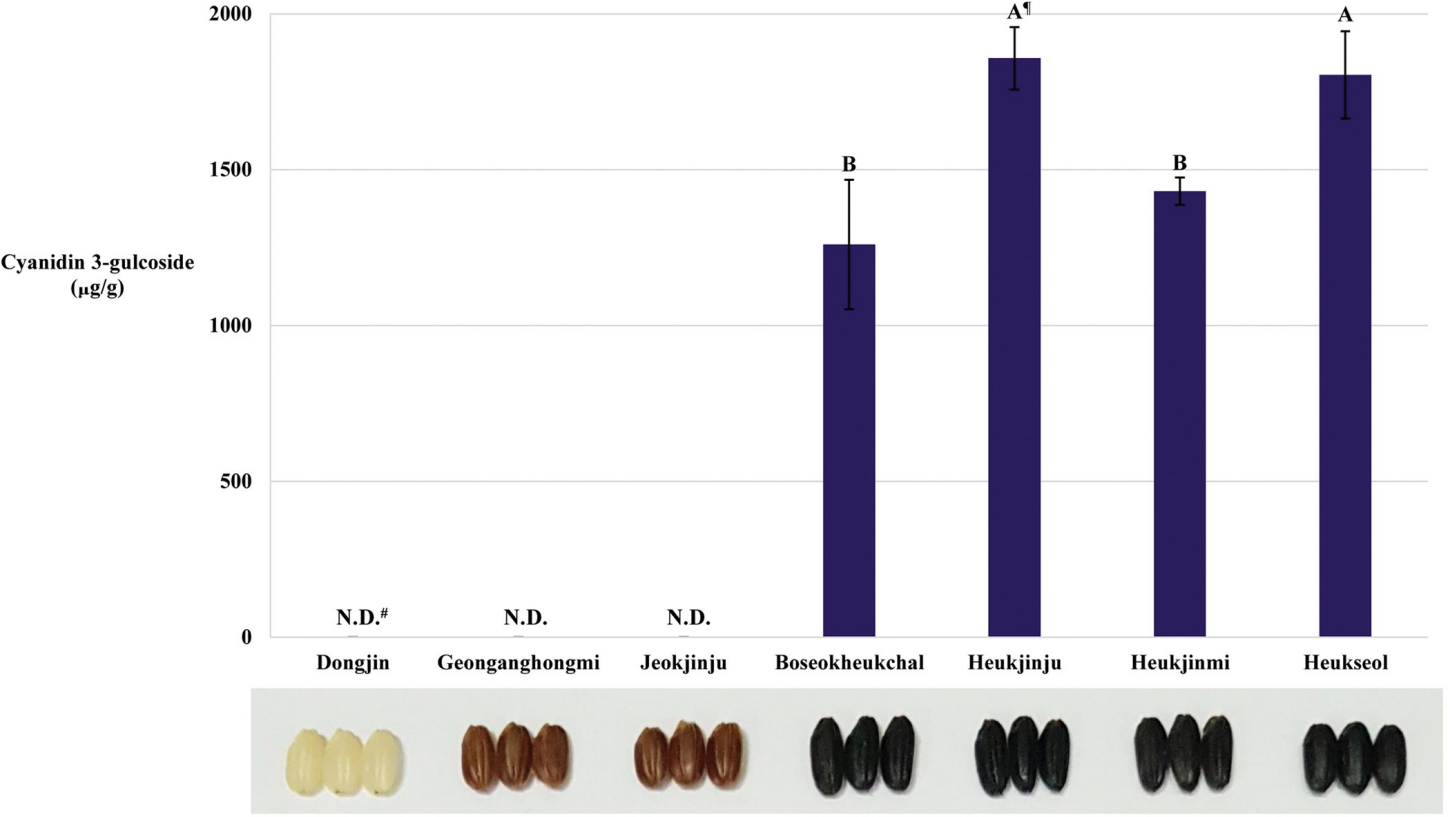
Liquid chromatography-mass spectrometry/mass spectrometry analysis of cyanidin 3-glucoside in hulled seeds of pigmented rice cultivars. Dongjin, Geonganghongmi, Jeokjinju, Boseokheukchal, Heukjinju, Heukjinmi, and Heukseol. ^¶^: Duncan’s Multiple Range Test (DMRT, α = 0.05) was performed after the confirmation of statistical significance for these data through analysis of variance (ANOVA). ^#^N.D.: not detected. The data represents mean ± standard deviation (SD).

We quantified C3G from the hulled seeds of seven rice cultivars, including Dongjin, Geonganghongmi, Jeokjinju, Boseokheukchal, Heukjinju, Heukjinmi, and Heukseol, through liquid chromatography/mass spectrometry-mass spectrometry (LC/MS-MS) (Fig 2). Of seven rice cultivars used in this study, C3G was detected only in the hulled seeds of black rice cultivars. Based on the quantities of C3G, four black rice cultivars are statistically classified into two groups; Boseokheukchal and Heukjinmi; Heukjinju and Heukseol. The quantities of C3G in the hulled seeds of Heukjinju and Heukseol were significantly higher than those in the hulled seeds of Boseokheukchal and Heukjinmi (Fig 2).

### Genes, involved in the biosynthetic pathway of anthocyanins, detected in developing black rice seeds through RNA-seq

We confirmed that the quantity of C3G and P3G maximally accumulated in the seeds of Heuknam and Heukseol, black rice cultivars, at around 20 DAH (Lee *et al*., unpublished), which corresponds to a previous report [57], and collected developing seeds of Dongjin, Geonganghongmi, Jeokjinju, Boseokheukchal, Heukjinju, Heukjinmi, and Heukseol at 20 DAH for gene expression analysis. To check the overall expression patterns of genes putatively involved in the biosynthetic pathway of anthocyanins, including *bHLH* [21, 45], *MYB* [20, 58], *WD40* [59], *PAL* [22], *C4H* [23, 24], *4CL* [25], *CHS* [26, 27], *CHI* [28–30], *F3H* [31, 32], *F3*´*H* [36, 37], *DFR* [33–35], and *ANS* [39–41], and other genes indirectly related to the biosynthetic pathway of anthocyanins, including *HCT* [60], *CCR* [61, 62], *CAD* [63], and *LAR* [64], in developing seeds of white, red, and/or black rice at 20 DAH, only one replicate of total RNA samples of Dongjin, Jeokjinju, or Heukseol was chosen for RNA-seq through Illumina sequencing. These RNA-seq data were used to identify potential candidate genes involved in the biosynthetic pathway of anthocyanins.

We detected a total 34,290 genes in developing rice seeds of Dongjin, Jeokjinju and/or Heukseol at 20 DAH after analysis of raw data of RNA-seq (S2 Table) and, based on amino acid sequence homology with each reference gene, i.e. *bHLH* [21, 45], *MYB* [20, 58], *WD40* [59], *PAL* [22], *C4H* [23, 24], *4CL* [25], *CHS* [26, 27], *CHI* [28–30], *F3H* [31, 32], *F3*´*H* [36, 37], *DFR* [33–35], *ANS* [39–41], *HCT* [60], *CCR* [61, 62], *CAD* [63], and *LAR* [64], searched and selected orthologous gene(s) for each reference gene (S2 and S5 Tables). For these candidate genes, the verification was intensively performed through qRT-PCR with more than three biological replicates.

Fifty-four bHLH genes and seventy-five MYB genes were detected in developing seeds of Dongjin, Jeokjinju and/or Heukseol at 20 DAH (S5 Table). Among these genes, three *bHLH*s—*LOC_Os01g09990*, *LOC_Os04g47040*, and *LOC_Os04g47059* (known as *OSB2* or *Kala4*)—and three *MYB*s—*LOC_Os01g49160*, *LOC_Os01g74410*, and *LOC_Os03g29614*—only showed preferential upregulation in developing seeds of Heukseol, compared to those of Dongjin and Jeokjinju. Moreover, of homologs for each gene, we could confirm that *WD40* (*LOC_Os02g45810*), *CHS* (*LOC_Os11g32650*), *CHI* (*LOC_Os03g60509*), *F3H* (*LOC_Os04g56700*), *F3*´*H* (*LOC_Os10g17260*), *DFR* (*LOC_Os01g44260*), and *ANS* (*LOC_Os01g27490*) were also preferentially upregulated in developing seeds of Heukseol. Predominantly, *ANS* (*LOC_Os01g27490*) was only expressed in developing seeds of Heukseol, but not in those of Dongjin and Jeokjinju (S5 Table).

Of these genes in S5 Table, based on their preferential expression patterns in the developing seeds of Heukseol, a black rice cultivar, we selected genes putatively involved in the biosynthetic pathway of anthocyanins, i.e., *bHLH1*, *LOC_Os04g47059*; *bHLH2*, *LOC_Os04g47040*; *MYB*, *LOC_Os01g49160*; *WD40*, *LOC_Os02g45810*; *PAL*, *LOC_Os02g41630*; *C4H*, *LOC_Os05g25640*; *4CL*, *LOC_Os02g08100*; *HCT*, *LOC_Os04g42250*; *CCR*, *LOC_Os09g25150*; *CAD*, *LOC_Os02g09490*; *CHS*, *LOC_Os11g32650*; *CHI*, *LOC_Os03g60509*; *F3H*, *LOC_Os04g56700*; *F3*´*H*, *LOC_Os10g17260*; *DFR*, *LOC_Os01g44260*; *ANS*, *LOC_Os01g27490*; and *LAR*, *LOC_Os03g15360*, to verify their expression in developing seeds of Dongjin, Geonganghongmi, Jeokjinju, Boseokheukchal, Heukjinju, Heukjinmi, and Heukseol at 20 DAH by qRT-PCR (S1 Fig and S5 Table). As mentioned above, these genes, except for *4CL* (*LOC_Os02g08100*), *HCT* (*LOC_Os04g42250*), *CCR* (*LOC_Os09g25150*), *CAD* (*LOC_Os02g09490*), and *LAR* (*LOC_Os03g15360*), showed preferential upregulation in developing seeds of Heukseol, compared to those of Dongjin and Jeokjinju (S1 Fig and S5 Table). We further investigated the expression levels of genes related to the biosynthesis of anthocyanins from RNA-seq data of Nipponbare (white rice) in the Rice Genome Annotation Project (http://rice.uga.edu/) to support our RNA-seq data (S2 Fig). As shown in developing seeds of Dongjin (S1 Fig and S5 Table), several genes, including *bHLH*s (*LOC_Os04g47040* and *LOC_Os04g47059*), *MYB* (*LOC_Os01g49160*), *WD40* (*LOC_Os02g45810*), *CHS* (*LOC_Os11g32650*), *CHI* (*LOC_Os03g60509*), *F3H* (*LOC_Os04g56700*), *F3*´*H* (*LOC_Os10g17260*), *DFR* (*LOC_Os01g44260*), and *ANS* (*LOC_Os01g27490*), upregulated in developing seeds of Heukseol, a black rice cultivar, were not upregulated in Nipponbare seed samples, including seeds at ten days after pollination (DAP), the embryo at 25 DAP and endosperm at 25 DAP, but, in Nipponbare 5 DAP seeds, *MYB* (*LOC_Os01g49160*), *WD40* (*LOC_Os02g45810*), *F3H* (*LOC_Os04g56700*), and *DFR* (*LOC_Os01g44260*) were up-regulated, compared to other genes (S2 Fig). *bHLH1* (*LOC_Os04g47059*) was upregulated only in seedling_leaf and anther of Nipponbare, and *ANS* (*LOC_Os01g27490*) was upregulated in anther (S2 Fig).

### Verification of the expression of genes involved in the biosynthetic pathway of anthocyanins in RNA-seq data of developing black rice seeds

In developing seeds of Dongjin, Geonganghongmi, Jeokjinju, Boseokheukchal, Heukjinju, Heukjinmi, and Heukseol at 20 DAH, we verified the expression of genes in the phenylpropanoid pathway, i.e., *PAL* (*LOC_Os02g41630*) [22], *C4H* (*LOC_Os05g25640*) [23, 24], *4CL* (*LOC_Os02g08100*) [25], *HCT* (*LOC_Os04g42250*) [60], *CCR* (*LOC_Os09g25150*) [61, 62], and *CAD* (*LOC_Os02g09490*) [63] through qRT-PCR (Fig 1, S3 Fig and S4 Table). These genes were classified into two groups: *PAL*, *C4H*, and *4CL*, which biosynthesize *p*-coumaroyl CoA, an intermediate in phenylpropanoid pathway [65] and a precursor in the biosynthetic pathway of flavonoids [26, 27], including anthocyanins; *HCT*, *CCR*, and *CAD*, genes in a branching point from *p*-coumaroyl CoA toward the biosynthetic pathway of monolignols [60, 62, 63]. As a result of the expression analysis for these genes in the phenylpropanoid pathway, we did not identify any apparent black rice-specific expression patterns in developing seeds at 20 DAH (S3 Fig).

**Fig 3:**
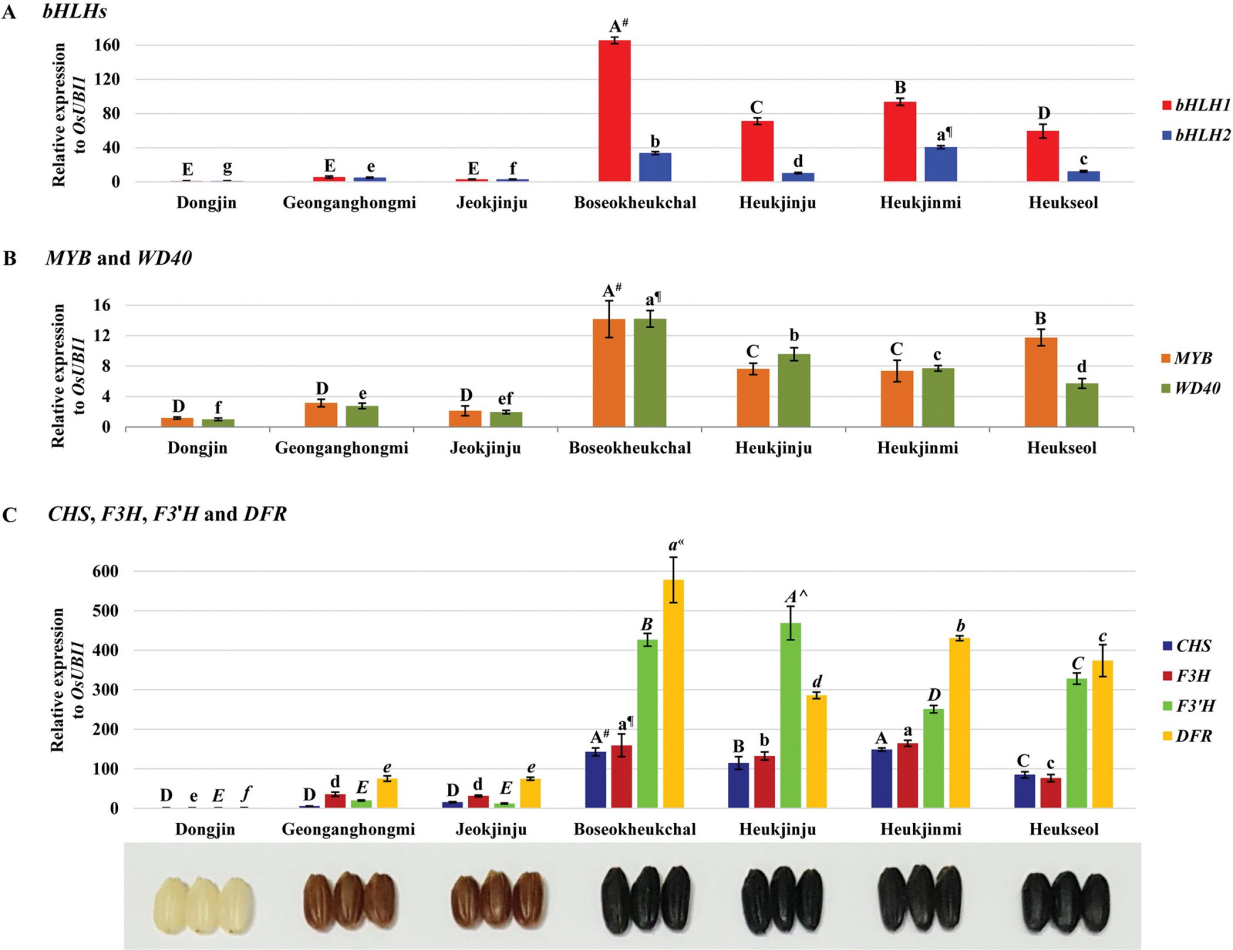
The relative expression of differentially upregulated genes in the biosynthetic pathway of anthocyanins in developing seeds of black rice cultivars, Boseokheukchal, Heukjinju, Heukjinmi, and Heukseol at twenty days after heading through q quantitative real-time polymerase chain reaction. **A.** *bHLH1*: *LOC_Os04g47059*, *bHLH2*: *LOC_Os04g47040*; **B.** *MYB*: *LOC_Os01g49160*, *WD40*: *LOC_Os02g45810*; **C.** *CHS*: *LOC_Os11g32650*, *F3H*: *LOC_Os04g56700*, *F3’H*: *LOC_Os10g17260*, *DFR*: *LOC_Os01g44260*. The data represents mean ± standard deviation (SD). ^#^, ^¶^, ^, ^«^: Duncan’s Multiple Range Test (DMRT, α = 0.05) was performed after the confirmation of statistical significance for each dataset through analysis of variance (ANOVA).

Putative regulator genes, including *bHLH1* (*LOC_Os04g47059*), *bHLH2* (*LOC_Os04g47040*), *MYB* (*LOC_Os01g49160*), and *WD40* (*LOC_Os02g45810*), and structural genes, including *CHS* (*LOC_Os11g32650*), *F3H* (*LOC_Os04g56700*), *F3*´*H* (*LOC_Os10g17260*), and *DFR* (*LOC_Os01g44260*), in the biosynthetic pathway of anthocyanins were explicitly upregulated in black rice seeds at 20 DAH (Fig 3). Especially, of two *bHLH* genes, *bHLH1* (*LOC_Os04g47059*) showed much higher relative expression in black rice seeds than *bHLH2* (*LOC_Os04g47040*). Although for these genes, Geonganghongmi and Jeokjinju, red rice cultivars, exhibited significantly lower expression levels in seeds at 20 DAH than those of black rice cultivars, these red rice cultivars had much higher expression levels in their seeds, compared to Dongjin, a white rice cultivar. Moreover, four genes, including *bHLH2* (*LOC_Os04g47040*), *WD40* (*LOC_Os02g45810*), *F3H* (*LOC_Os04g56700*) and *DFR* (*LOC_Os01g44260*), showed statistically significant upregulation in the seeds of red rice than those of white rice (Fig 3). Interestingly, *CHI* (*LOC_Os03g60509*), located between *CHS* (*LOC_Os11g32650*) and *F3H* (*LOC_Os04g56700*) in the biosynthetic pathway of anthocyanins [26–32], did not exhibit black rice-specific upregulation in its expression but seemed to have significantly higher expression in the seeds of red and black rice cultivars than those of white rice (S4 Fig).

**Fig 4:**
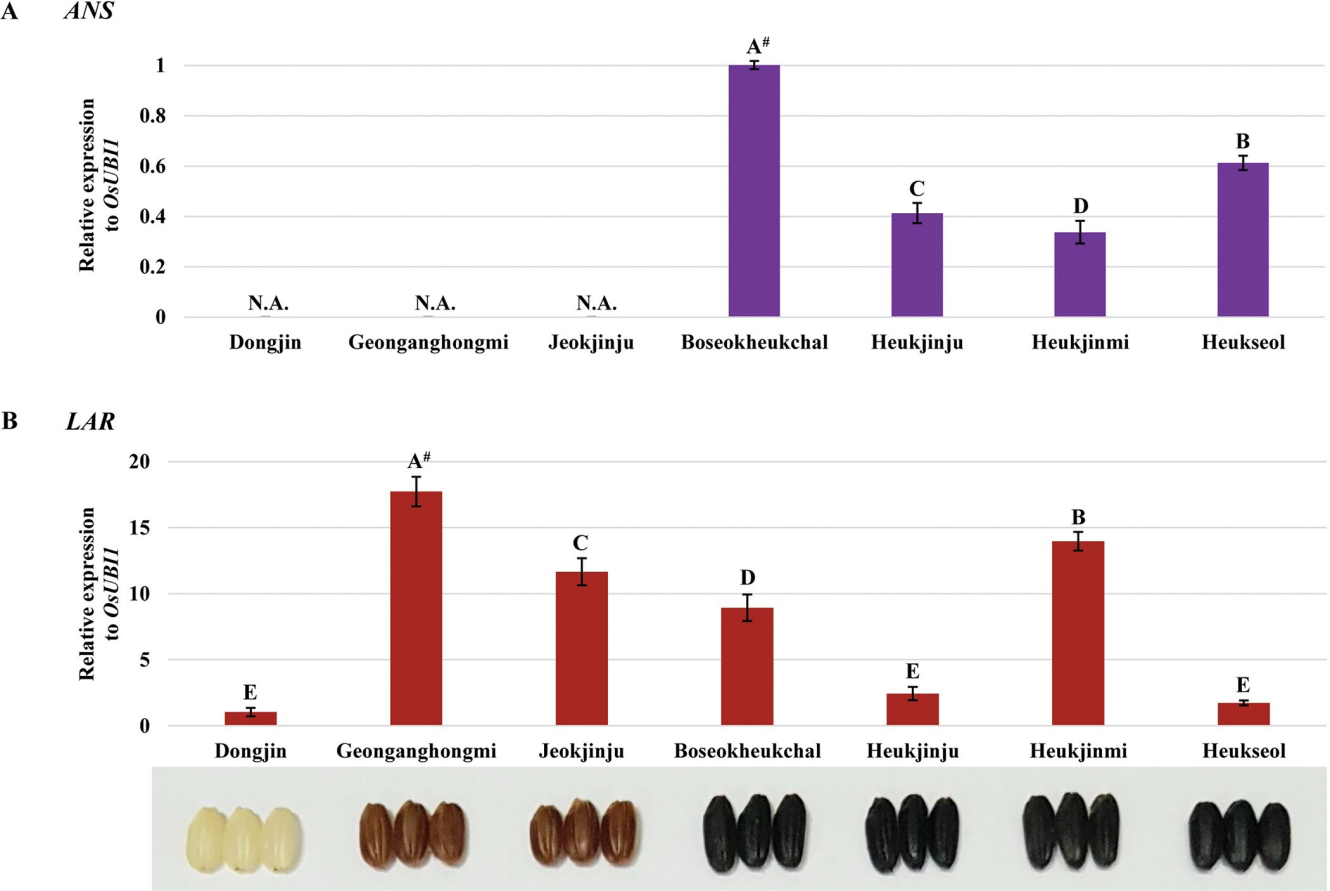
Relative expression of *ANS* (A, *LOC_Os01g27490*) and *LAR* (B, *LOC_Os03g15360*), respectively, in developing seeds of Dongjin, Geonganghongmi, Jeokjinju, Boseokheukchal, Heukjinju, Heukjinmi and Heukseol at twenty days after heading through a quantitative real-time polymerase chain reaction. The data represents mean ± standard deviation (SD). ^#^: Duncan’s Multiple Range Test (DMRT, α = 0.05) was performed after the confirmation of statistical significance for each dataset through analysis of variance (ANOVA). N.A.: not applicable.

We verified that *ANS* (*LOC_Os01g27490*) showed black rice-specific expression patterns in seeds at 20 DAH, but it showed no expression in white rice (Dongjin) and red rice (Geonganghongmi and Jeokjinju) seeds (Fig 4), as shown in S5 Table. Among black rice cultivars, there were statistical differences in the expression of *ANS* (*LOC_Os01g27490*) (Fig 4). Moreover, we investigated the expression levels of *LAR* (*LOC_Os03g15360*), which converts leucocyanidin into catechin, one of the red rice-specific compounds [64, 66], in developing seeds of Dongjin, Geonganghongmi, Jeokjinju, Boseokheukchal, Heukjinju, Heukjinmi, and Heukseol at 20 DAH, because Heukjinmi (black rice) has red rice as one of the parents as mentioned above. Of these rice cultivars, Geonganghongmi (red rice) has the maximum expression of *LAR* (*LOC_Os03g15360*) in its seeds, and Jeokjinju (red rice), Boseokheukchal (black rice) and Heukjinmi (black rice) also showed statistically significant upregulation of *LAR* (*LOC_Os03g15360*) in their seeds, compared to Dongjin (white rice), Heukjinju (black rice), and Heukseol (black rice) (Fig 4).

### Correlation analysis between the quantity of cyanidin 3-glucoside and the expression level of each gene involved in the biosynthetic pathway of anthocyanins in developing rice seeds

**Fig 5:**
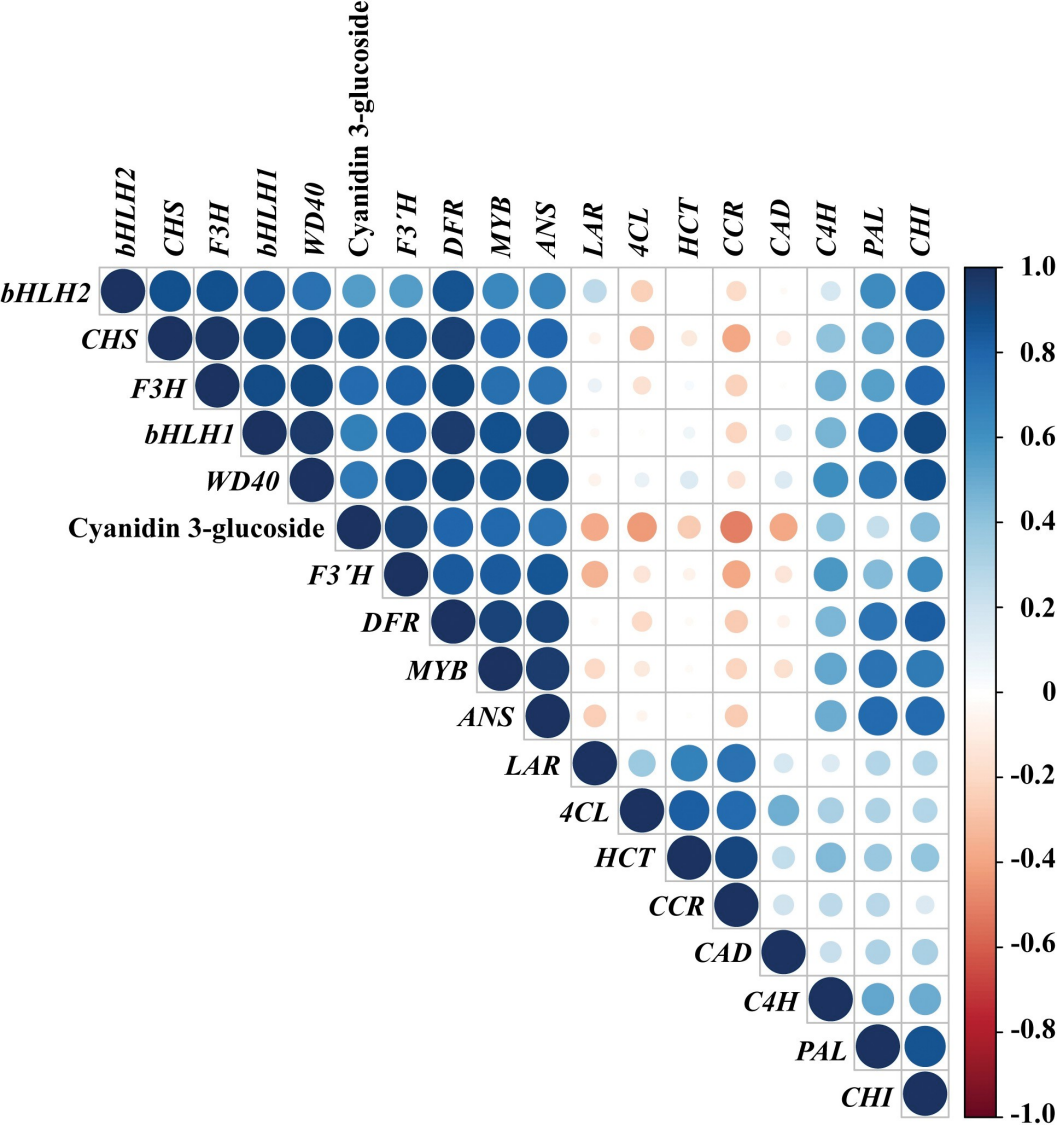
Correlation analysis in Dongjin, Geonganghongmi, Jeokjinju, Boseokheukchal, Heukjinju, Heukjinmi, and Heukseol, between the quantity of cyanidin 3-glucoside in hulled rice seeds and the expression level of each gene involved in the biosynthetic pathway of anthocyanins in seeds at twenty days after heading, and expression level of these genes. Bigger circles indicate more statistical significance. Blue and red colors show a positive and negative correlation, respectively.

We performed a correlation analysis in Dongjin, Geonganghongmi, Jeokjinju, Boseokheukchal, Heukjinju, Heukjinmi, and Heukseol, to investigate the statistical relationship in the quantity of C3G in hulled rice seeds and the expression levels of each gene putatively involved in the biosynthetic pathway of anthocyanins in rice seeds at 20 DAH (Fig 5 and S6 Table). The quantity of C3G in hulled rice seeds is positively correlated to the expression of these genes, including *bHLH1* (*LOC_Os04g47059*), *bHLH2* (*LOC_Os04g47040*), *MYB* (*LOC_Os01g49160*), *WD40* (*LOC_Os02g45810*), *CHS* (*LOC_Os11g32650*), *F3H* (*LOC_Os04g56700*), *F3*´*H* (*LOC_Os10g17260*), *DFR* (*LOC_Os01g44260*), and *ANS* (*LOC_Os01g27490*), preferentially upregulated in black rice seeds as described in Figs 3 and 4. These genes also had a positive correlation between their expression values. Interestingly, the expression of *CHI* (*LOC_Os03g60509*) did not correlate with the quantity of C3G. However, it positively correlated with the expression of genes preferentially upregulated in black rice seeds mentioned above. In addition, the expression of *CCR* (*LOC_Os09g25150*), which shares *p*-coumaroyl CoA as a precursor with *CHS* (*LOC_Os11g32650*) [26, 27, 61, 62], was negatively correlated with the quantity of C3G (Fig 5 and S6 Table).

## Discussion

In rice, C3G and P3G were reported as major and minor anthocyanins, respectively [8, 10, 46], and anthocyanins were differentially biosynthesized in different black rice cultivars [46]. As Kim et al. described [46], any detectable C3G was not identified in seeds of Dongjin, a white rice cultivar, and Geonganghongmi and Jeokjinju, red rice cultivars, and, in black rice, two groups showed statistically different quantities of C3G (Fig 2).

As reported in maize, the biosynthesis of anthocyanins was regulated by two transcription factors: *R*, a *bHLH* gene, and *C1*, a myb gene [16–19]. The overexpression of *C1* and *B-Peru*, a *bHLH* gene, resulted in the biosynthesis of anthocyanins in developing white rice seeds [21]. Furthermore, the overexpression of maize *C1* and rice *bHLH* gene [*OSB1* (AB021079, *LOC_Os04g47080*) or *OSB2* (AB021080, *LOC_Os04g47059*)] resulted in the accumulation of anthocyanins in developing white rice seeds. However, no anthocyanin accumulation occurred in developing rice seeds upon the overexpression of only one gene of *C1*, *B-Peru*, *OSB1*, or *OSB2*. These results suggested that, in developing rice seeds, the overexpression of *R*, *bHLH* gene, and *C1*, myb gene, is essential for the biosynthesis of anthocyanins [21]. The *Kala4* (*LOC_Os04g47059*), an essential *bHLH* gene involved in the biosynthesis of anthocyanins in rice seeds, was upregulated in developing rice seeds more than in those white rice seeds [45]. The overexpression of *Kala4* led to the accumulation of anthocyanins in near-isogenic rice lines with *Kala3*, a myb gene functionally expressed and without *Kala4* being functionally expressed [45, 67]. In our RNA-seq data at 20 DAH rice seeds, we identified fifty-four *bHLH*s and seventy-five *MYB*s, and, of them, three *bHLH*s, including *LOC_Os01g09990*, *LOC_Os04g47040* (*bHLH2*), and *LOC_Os04g47059* (*bHLH1*; known as *OSB2* or *Kala4*), and 3 *MYB*s, including *LOC_Os01g49160* (*MYB*), *LOC_Os01g74410*, and *LOC_Os03g29614*, were differentially upregulated in developing black rice seeds. This indicated that those genes are putatively involved in the biosynthesis of anthocyanins in developing seeds of black rice (S5 Table). Interestingly, the *MYB* gene (Y15219, *LOC_Os06g10350*) homologous to maize *C1* reported by Reddy et al. did not show black rice-specific expression patterns in developing seeds [20], thereby indicating that there are other putative functional *MYB*s, involved in the biosynthesis of anthocyanins, with seed-specific expression patterns (S5 Table).

In addition, *WD40* (*LOC_Os02g45810*) was reported to regulate the biosynthesis of anthocyanins in *Arabidopsis thaliana* [59] and rice [68] and was differentially upregulated in black rice seeds (Fig 3B). However, in contrast to *bHLH* and *MYB*, *WD40* might be functionally expressed in developing white rice seeds because Sakamoto et al. showed the accumulation of anthocyanins in seeds of white rice by the overexpression of *bHLH* and *MYB*, but not with WD40 [21].

The structural genes, including *CHS* (*LOC_Os11g32650*), *F3H* (*LOC_Os04g56700*), *F3*´*H* (*LOC_Os10g17260*), *DFR* (*LOC_Os01g44260*), and *ANS* (*LOC_Os01g27490*), involved in the biosynthetic pathway of anthocyanins, were differentially upregulated in developing black rice seeds (S1 Fig), compared to the expression data in Nipponbare, a white rice cultivar, with no such upregulation as mentioned above (S2 Fig). Further verification of RNA-seq data by qRT-PCR exhibited that, of the structural genes in the biosynthetic pathway of anthocyanins, *CHS* (*LOC_Os11g32650*), *F3H* (*LOC_Os04g56700*), *F3*´*H* (*LOC_Os10g17260*), and *DFR* (*LOC_Os01g44260*) were differentially upregulated in developing seeds of black rice (Fig 3C). Moreover, *ANS* (*LOC_Os01g27490*), which converts leucoanthocyanidin into anthocyanidin [39–41], was expressed only in developing seeds of black rice but not in seeds of white and red rice (Fig 4A and S5 Table). However, there are remaining questions about the switch-on system for expressing *ANS* in black rice seeds, and it is required to carry out further investigations for this issue.

Catechin is converted from leucocynidin, a precursor shared by *LAR* [64] and *ANS* [39–41], by the reaction of *LAR* [64], and procyanidins were polymerized from catechin [64]. They were biosynthesized in seeds of red rice but not in white and black rice seeds [66]. As mentioned above, the expression of *LAR* (*LOC_Os03g15360*) was significantly upregulated in the seeds of Heukjinmi, which was crossed with red rice, as in the seeds of red rice cultivars, Geonganghongmi and Jeokjinju (Fig 4B). However, it was also significantly upregulated in the seeds of Boseokheukchal, compared to Dognjin (white rice) and the other two black rice cultivars, Heukjinju and Heukseol, indicating that higher expression of *LAR* is putatively related to the reduction of C3G biosynthesized in black rice seeds (Figs 2 and 4B). For Boseokheukchal, it is necessary to investigate whether red rice is incorporated as a parent. These expression analysis data are closely related to the C3G quantities in four black rice cultivars, divided into the first group of Heukjinju and Heukseol with significantly higher C3G content, and the second group of Boseokheukchal and Heukjinmi with significantly lower C3G content (Fig 2).

Furthermore, correlation analysis between C3G contents in hulled rice and the expression level of genes involved in the biosynthesis of anthocyanins revealed that the quantities of anthocyanins biosynthesized in black rice seeds are positively correlated to the expression level of *bHLH1* (*LOC_Os04g47059*), *bHLH2* (*LOC_Os04g47040*), *MYB* (*LOC_Os01g49160*), *WD40* (*LOC_Os02g45810*), *CHS* (*LOC_Os11g32650*), *F3H* (*LOC_Os04g56700*), *F3*´*H* (*LOC_Os10g17260*), *DFR* (*LOC_Os01g44260*), and *ANS* (*LOC_Os01g27490*), respectively (Fig 5 and S6 Table). However, there is still little doubt about the biosynthesis of anthocyanins in rice seeds because we carried out this study with limited information and just a few black rice cultivars. Therefore, with more black rice cultivars and with much deeper details of transcriptomic and genomic data, further studies are required to perfectly understand the biosynthesis of anthocyanins in developing seeds of black rice, thereby will resulting in establishment of database for gene expression of each gene related to the biosynthetic pathway of anthocyanins in developing seeds in various black rice cultivars. Furthermore, the results from further studies can efficiently and powerfully be utilized in rice breeding programs to improve the anthocyanin content in seeds. In addition, as shown in Figs 3 and 4, red rice cultivars exhibited very unique expression data, compared to those in white and black rice cultivars. Further study is also needed for more understanding of the biosynthetic pathway of proanthocyanidins in developing seeds of red rice through transcriptomic and genomic analysis tools.

## Conclusion

In this study, we elucidated that the C3G contents biosynthesized in black rice seeds positively correlate to the expression levels of genes, including *bHLH1*, *bHLH2*, *MYB*, *WD40*, *CHS*, *F3H*, *F3*´*H*, *DFR*, and *ANS*. In addition, compared to those of white and red rice cultivars, several genes that regulate the biosynthesis of anthocyanins, including *bHLH*s, *MYB*s, and *WD40*, were highly upregulated in developing seeds of black rice cultivars, and the structural genes in the biosynthetic pathway of anthocyanins, including *CHS*, *F3H*, *F3*´*H*, *DFR*, and *ANS*, were also differentially upregulated in black rice seeds. Moreover, we report new candidate transcription factor genes, *bHLH2* (*LOC_Os04g47040*), and *MYB*s (*LOC_Os01g49160*, *LOC_Os01g74410*, and *LOC_Os03g29614*), with black rice seed-specific expression patterns, for the biosynthesis of anthocyanins in black rice seeds.

## Supporting information

S1 Fig The relative expression of genes involved in the phenylpropanoid pathway and biosynthetic pathway of anthocyanins, detected in the seeds of Dongjin, Jeokjinju, and Heukseol at twenty days after heading through RNA-seq. *bHLH*: basic helix-loop-helix gene; *MYB*: myb gene; *WD40*: tryptophan-aspartic acid repeat protein gene; *PAL*: *phenylalanine ammonia-lyase*; *C4H*: *cinnamate 4-hydroxylase*; *4CL*: *4-coumarate*: *CoA ligase*; *HCT*: *hydroxycinnamoyl-CoA shikimate/quinate hydroxycinnamoyltransferase*; *CCR*: *cinnamoyl-CoA reductase*; *CAD*: *cinnamyl alcohol dehydrogenase*; *CHS*: *chalcone synthase*; *CHI*: *chalcone isomerase*; *F3H*: *flavanone 3-hydroxylase*; *DFR*: *dihydroflavonol 4-reductase*; *F3´H*: *flavonoid 3´-hydroxylase*; *ANS*: *anthocyanidin synthase*; and *LAR*: *leucoanthocyanidin reductase*. The scale bar indicates the normalized Log_2_ ratio (individual value/average value).
(TIF)

S2 Fig The relative expression of genes, involved in the phenylpropanoid pathway and biosynthetic pathway of anthocyanins, detected in Nipponbare (white rice) obtained from the RNA-seq data of the Rice Genome Annotation Project (http://rice.plantbiology.msu.edu/). *: days after pollination. The scale bar indicates the normalized Log2 ratio (individual value/average value).
(TIF)

S3 Fig The relative expression of genes involved in the phenylpropanoid pathway in developing seeds of Dongjin, Geonganghongmi, Jeokjinju, Boseokheukchal, Heukjinju, Heukjinmi, and Heukseol at twenty days after heading through a quantitative real-time polymerase chain reaction. **A.** *PAL* (*LOC_Os02g41630*), *C4H* (*LOC_Os05g25640*), and *4CL* (*LOC_Os02g08100*); **B.** *HCT* (*LOC_Os04g42250*), *CCR* (*LOC_Os09g25150*), and *CAD* (*LOC_Os02g09490*). The data represents mean ± standard deviation (SD). ^#^, ^¶^, ^: Duncan’s Multiple Range Test (DMRT, α = 0.05) was performed after confirming statistical significance for these data through analysis of variance (ANOVA).
(TIF)

S4 Fig The relative expression of *CHI* (*LOC_Os03g60509*) in the seeds of Dongjin, Geonganghongmi, Jeokjinju, Boseokheukchal, Heukjinju, Heukjinmi, and Heukseol at twenty days after heading through a quantitative real-time polymerase chain reaction. The data represents mean ± standard deviation (SD). ^#^: Duncan’s Multiple Range Test (DMRT, α = 0.05) was performed after the confirmation of statistical significance for these data through analysis of variance (ANOVA).
(TIF)

S1 Table Basic information of raw data generated from RNA sequencing. (DOCX)

S2 Table The expression value of genes detected in seeds of Dongjin, Jeokjinju, and Heukseol, respectively, at twenty days after heading through RNA-seq analysis. (expression values of each gene was normalized by division of the expression values of *OsUBI1* of Dongjin, Jeokjinju, or Heukseol, respectively).
(XLSX)

S3 Table List of primer sets for a quantitative real-time polymerase chain reaction. (DOCX)

S4 Table Black rice cultivars developed at the National Institute of Crop Science, Republic of Korea. Retrieved from: http://www.nics.go.kr/api/breed.do?m=100000128&homepageSeCode=nics.
(DOCX)

S5 Table The genes, putatively involved in the biosynthetic pathway of anthocyanins, selected from RNA-seq data of seeds of Dongjin, Jeokjinju, and Heukseol, respectively, at twenty days after heading. (expression values of each gene was normalized by division of the expression values of *OsUBI1* of Dongjin, Jeokjinju, or Heukseol, respectively).
(XLSX)

S6 Table The correlation coefficient, in Dongjin, Geonganghongmi, Jeokjinju, Boseokheukchal, Heukjinju, Heukjinmi, and Heukseol, obtained from correlation analysis between the quantity of cyanidin 3-glucoside in hulled rice seeds and the expression levels of each gene involved in the biosynthetic pathway of anthocyanins in seeds at twenty days after heading and between the expression levels of these genes. (DOCX)
