# Supplementary figures and images for: Reinterpretation of anthocyanins biosynthesis in developing black rice seeds through gene expression analysis
Source: PLoS One. 2023 Jun 2;18(6):e0286539. doi: 10.1371/journal.pone.0286539 (PMC10237452; doi:10.1371/journal.pone.0286539)

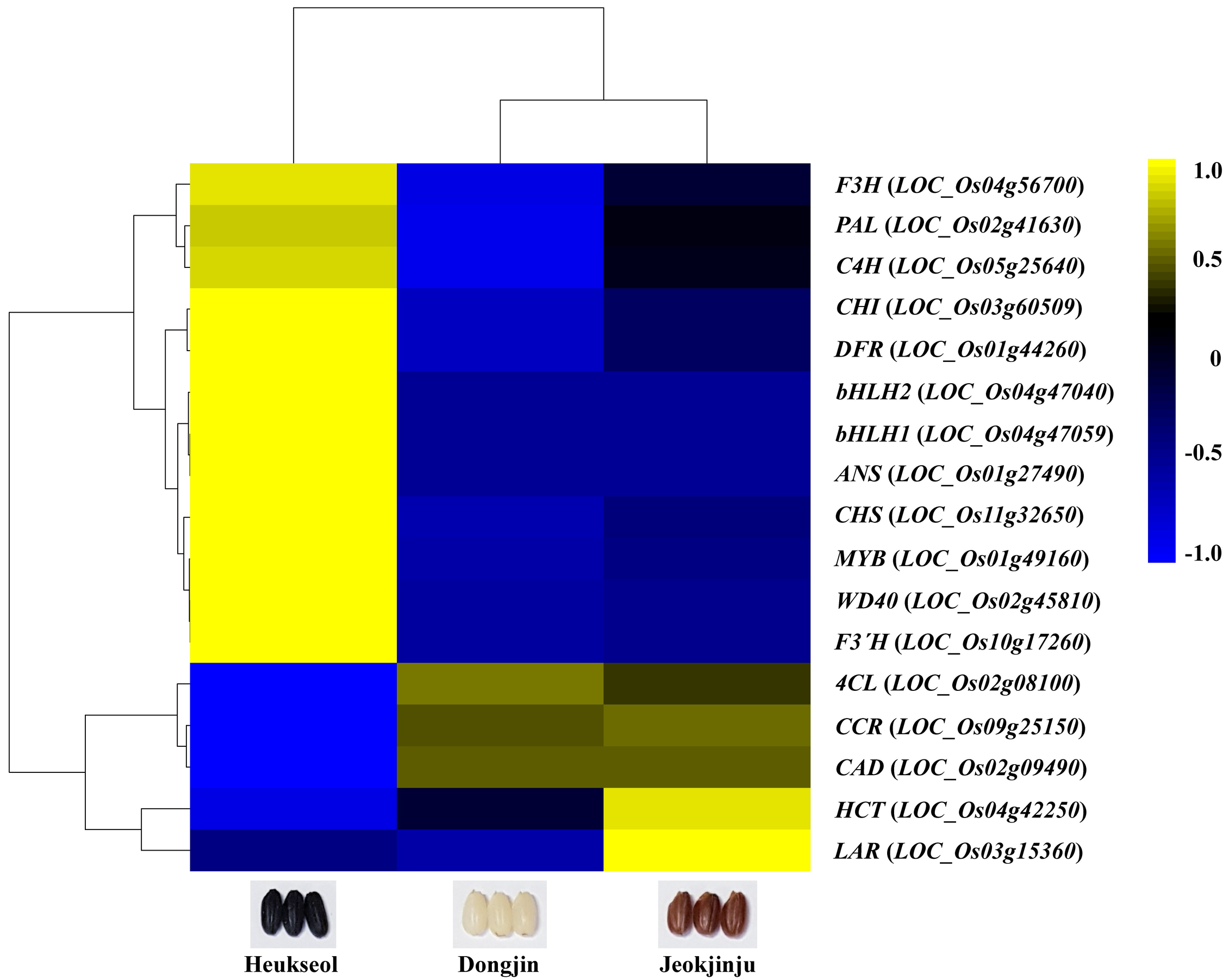

Supplement: S1 Fig — bHLH: basic helix-loop-helix gene; MYB: myb gene; WD40: tryptophan-aspartic acid repeat protein gene; PAL: phenylalanine ammonia-lyase; C4H: cinnamate 4-hydroxylase; 4CL: 4-coumarate: CoA ligase; HCT: hydroxycinnamoyl-CoA shikimate/quinate hydroxycinnamoyltransferase; CCR: cinnamoyl-CoA reductase; CAD: cinnamyl alcohol dehydrogenase; CHS: chalcone synthase; CHI: chalcone isomerase; F3H: flavanone 3-hydroxylase; DFR: dihydroflavonol 4-reductase; F3´H: flavonoid 3´-hydroxylase; ANS: anthocyanidin synthase; and LAR: leucoanthocyanidin reductase. The scale bar indicates the normalized Log2 ratio (individual value/average value). (TIF) [file pone.0286539.s001.tif]

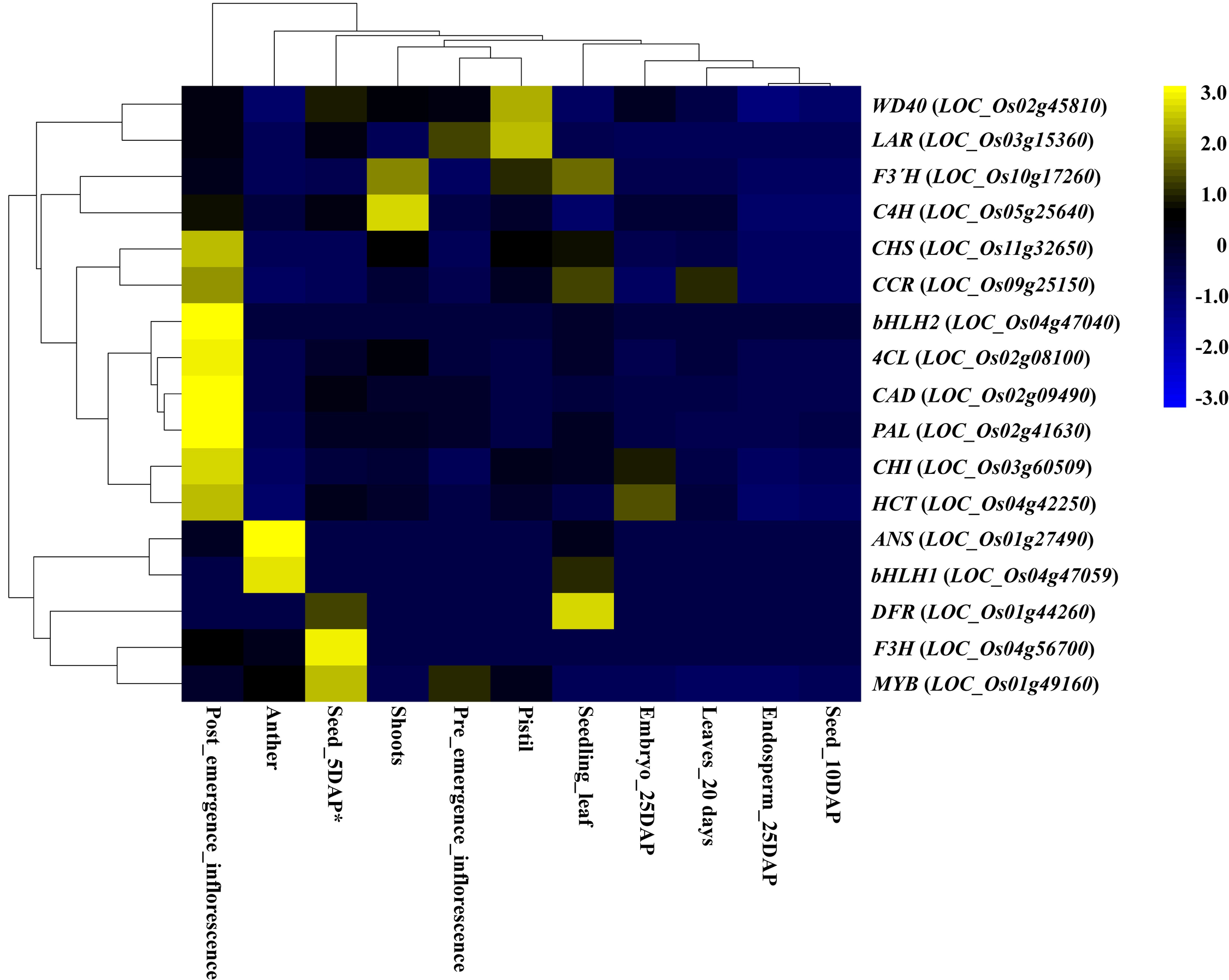

Supplement: S2 Fig — *: days after pollination. The scale bar indicates the normalized Log2 ratio (individual value/average value). (TIF) [file pone.0286539.s002.tif]

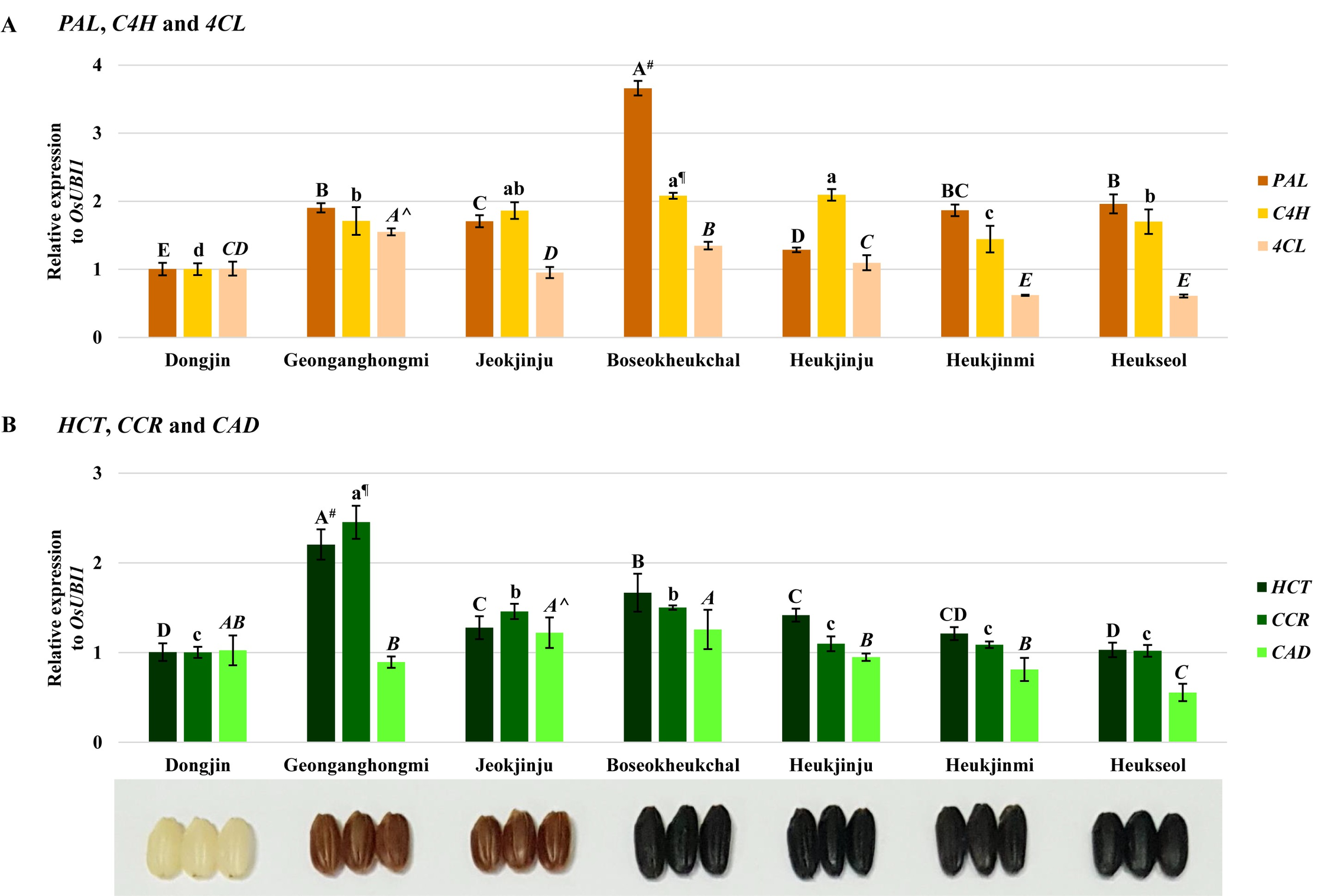

Supplement: S3 Fig — A. PAL (LOC_Os02g41630), C4H (LOC_Os05g25640), and 4CL (LOC_Os02g08100); B. HCT (LOC_Os04g42250), CCR (LOC_Os09g25150), and CAD (LOC_Os02g09490). The data represents mean ± standard deviation (SD). #, ¶, ^: Duncan’s Multiple Range Test (DMRT, α = 0.05) was performed after confirming statistical significance for these data through analysis of variance (ANOVA). (TIF) [file pone.0286539.s003.tif]

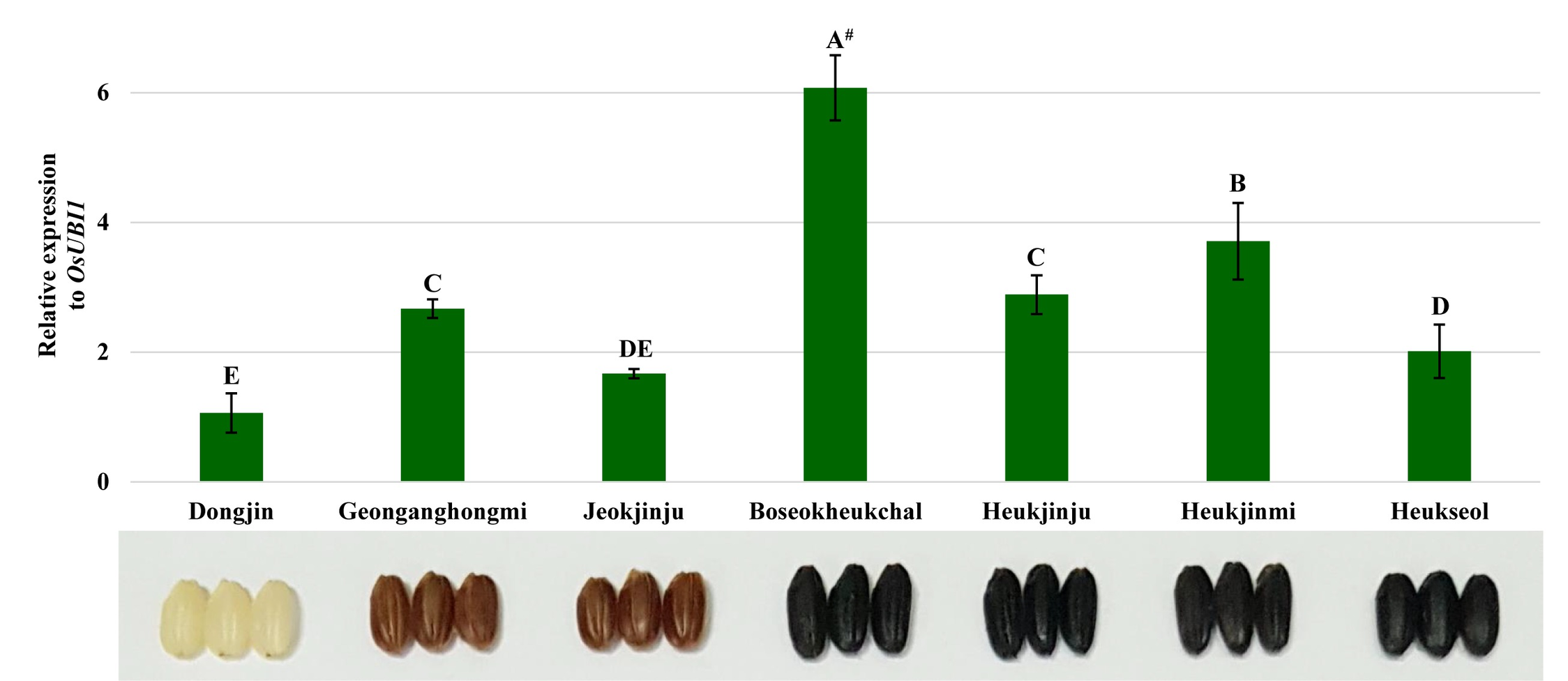

Supplement: S4 Fig — The data represents mean ± standard deviation (SD). #: Duncan’s Multiple Range Test (DMRT, α = 0.05) was performed after the confirmation of statistical significance for these data through analysis of variance (ANOVA). (TIF) [file pone.0286539.s004.tif]
