# Supplementary material for: Reinterpretation of anthocyanins biosynthesis in developing black rice seeds through gene expression analysis
Source: PLoS One. 2023 Jun 2;18(6):e0286539. doi: 10.1371/journal.pone.0286539 (PMC10237452; doi:10.1371/journal.pone.0286539)
Supplement: S1 Table — (DOCX) [file pone.0286539.s005.docx]

**S1 Table. Basic information of raw data generated from RNA sequencing.**

| Sample name | Total reads number | Sequence length (bp) | GC (%) | Mapping rate to MSU7 (%) |
| --- | --- | --- | --- | --- |
| Dongjin | 59,481,720 | 101 | 48.5 | 98.82 |
| Jeokjinju | 79,696,304 | 101 | 49 | 98.89 |
| Heukseol | 77,227,876 | 101 | 49.54 | 98.51 |
