# Supplementary material for: Reinterpretation of anthocyanins biosynthesis in developing black rice seeds through gene expression analysis
Source: PLoS One. 2023 Jun 2;18(6):e0286539. doi: 10.1371/journal.pone.0286539 (PMC10237452; doi:10.1371/journal.pone.0286539)
Supplement: S3 Table — (DOCX) [file pone.0286539.s007.docx]

**S3 Table. List of primer sets for a quantitative real-time polymerase chain reaction.**

| Gene name (Gene ID) | PCR Product size  (bp) | Forward (5' → 3') | Reverse (5' → 3') | Annealing T.(°C) |
| --- | --- | --- | --- | --- |
| *bHLH1* (*LOC_Os04g47059*) | 160 | GAAGTCACTACTCCCCTCCGTCC | CGCCTTCCCCTGTCCAAT | 60 |
| *bHLH2* (*LOC_Os04g47040*) | 160 | GAGTGTTCGACGCGATCAAGA | CCACCGATAGCTTTCTGGAGAG | 60 |
| *MYB* (*LOC_Os01g49160*) | 160 | ATCGCGCTCCCGCAGAAAG | TGGACCACCTGCTTCCGATG | 59.5 |
| *WD40* (*LOC_Os02g45810*) | 160 | CAAGCGCTGATTTGGGAGCTG | GCTGGACCTTGTTCTCAAAGGC | 58.5 |
| *PAL* (*LOC_Os02g41630*) | 160 | GTTCCCGCTCTACCGCTTC | GCCGTTCCACTCCTTGAGG | 57 |
| *C4H* (*LOC_Os05g25640*) | 160 | TCGCGCTGCCCATCAT | GCGTCGATGGGCTTGC | 58.5 |
| *4CL* (*LOC_Os02g08100*) | 160 | CGACCAAGAACACCATCGAC | GTGATGAGCAGCGCCTCC | 58 |
| *HCT* (*LOC_Os04g42250*) | 160 | GATTTTGGATGGGGAAGACCA | CACCTCGAAGATCAGCTTCCTG | 58 |
| *CCR* (*LOC_Os09g25150*) | 160 | GGTGAACCCACGGAAGCA | CAACTCTTCTGGGATCTCTTTGCT | 58 |
| *CAD* (*LOC_Os02g09490*) | 160 | CGCGAGTGCCATCCGT | CTAGCCCCGCCGGGAT | 59 |
| *CHS* (*LOC_Os11g32650*) | 160 | GCGCCACGTGCTGTCC | CTCAACGGTGAGGCCGG | 58.5 |
| *CHI* (*LOC_Os03g60509*) | 160 | GGCGCTGGCCAAGAAGTG | GTTCTCCGTCACCTTGTCCGA | 58 |
| *F3H* (*LOC_Os04g56700*) | 160 | CCGGCGCAAGATGGC | GGCAAGAATCTCGTCGAGAGAC | 60 |
| *F3'H* (*LOC_Os10g17260*) | 160 | CTGATGACTGCCACGCTAGTG | CTCCATAAGCCGATGGAAGC | 58.5 |
| *DFR* (*LOC_Os01g44260*) | 160 | CCGGACGTGCAGGGAG | GACCAACGCTTCTGTTTCAGC | 60 |
| *ANS* (*LOC_Os01g27490*) | 160 | AGTACGTCCCCGTGTCGC | TGAAGGAGCAGGTCGTCGTC | 58 |
| *LAR* (*LOC_Os03g15360*) | 160 | CCGGAGAGCATAGTCGCTTCA | GGACATGTATGTAGTCGTCGAAGCA | 60 |
| *UBI* (*LOC_Os03g13170*) | 472 | TGAAGACCCTGACTGGGAAG | CACGGTTCAACAACATCCAG | 59 |

*bHLH*: basic helix-loop-helix gene; *MYB*: myb gene; *WD40*: tryptophan-aspartic acid repeat protein gene; *PAL*: *phenylalanine ammonia-lyase*; *C4H*: *cinnamate 4-hydroxylase*; *4CL*: *4-coumarate: CoA ligase*; *HCT*: *hydroxycinnamoyl-CoA shikimate/quinate hydroxycinnamoyltransferase*; *CCR*: *cinnamoyl-CoA reductase*; *CAD*: *cinnamyl alcohol dehydrogenase*; *CHS*: *chalcone synthase*; *CHI*: *chalcone isomerase*; *F3H*: *flavanone 3-hydroxylase*; *DFR*: *dihydroflavonol 4-reductase*; *F3´H*: *flavonoid 3´-hydroxylase*; *ANS*: *anthocyanidin synthase*; *LAR*: *leucoanthocyanidin reductase*; *UBI*: *UBIQUITIN*, *OsUBI1*.
