# Supplementary material for: Reinterpretation of anthocyanins biosynthesis in developing black rice seeds through gene expression analysis
Source: PLoS One. 2023 Jun 2;18(6):e0286539. doi: 10.1371/journal.pone.0286539 (PMC10237452; doi:10.1371/journal.pone.0286539)
Supplement: S4 Table — Retrieved from: http://www.nics.go.kr/api/breed.do?m=100000128&homepageSeCode=nics. (DOCX) [file pone.0286539.s008.docx]

**S4 Table. Black rice cultivars developed at the National Institute of Crop Science, Republic of Korea.**

Retrieved from: http://www.nics.go.kr/api/breed.do?m=100000128&homepageSeCode=nics.

| Cultivars | Release year | Parent(s) for black rice | Anthocyanin (or cyanidin 3-glucoside)  content in seeds | Cultivar for control;  Anthocyanin (or cyanidin 3-glucoside)  content in seeds |
| --- | --- | --- | --- | --- |
| Heuknam | 1997 | Shanghai xiang xue nuo | N.A.* | N.A. |
| Heukjinju | 1998 | Longjin1 | 6,556 ppm^ (anthocyanin^$^) | Heukjinmi; 2,922 ppm (anthocyanin^$^) |
| Heukhyang | 2000 | SX864 | N.A. | N.A. |
| Heukgwang | 2003 | Longjin1 | 532 ppm (cyanidin 3-glucoside) | Heuknam; 306 ppm (cyanidin 3-glucoside) |
| Josaengheukchal | 2004 | Dong Bei nuo 149, SX864 | N.A. | N.A. |
| Heukseol | 2007 | Longjin1 | 813 me/100g^ (cyanidin 3-glucoside) | Heukjinju; 765 me/100g (cyanidin 3-glucoside) |
| Boseokheukchal | 2008 | Heukmi H31,  Shanghai xiang xue nuo | 717 mg/1000g^ (anthocyanin^$^) | Heukjinju; 717 mg/1000g (anthocyanin^$^) |
| Seonhyangheukmi | 2011 | Longjin1 | 136.4 mg/100g^ (anthocyanin^$^) | Heukjinju; 58.8 mg/100g (anthocyanin^$^) |
| Joenheukmi | 2011 | Dohoku 149, SX864 | 258 mg/100g (anthocyanin^$^) | Heukjinju; 204 mg/100g (anthocyanin^$^) |
| Heuksujeong | 2012 | SX864 | 8.4 mg/100g (anthocyanin^$^) | Heuknam; 16.0 mg/100g (anthocyanin^$^) |
| Nunkeunheukchal-1ho | 2014 | Dong Bei nuo 149, SX864 | 28.8 mg/g^ (anthocyanin^$^) | Josaengheukchal; 25.7 mg/g (anthocyanin^$^) |
| Cheonghyangheukmi | 2015 | Dong Bei nuo 149, SX864 | 108.4 mg/100g (anthocyanin^$^) | Heuknam; 89.2 mg/100g (anthocyanin^$^) |
| Heukjinmi^#^ | 2015 | Heukmi H31,  Shanghai xiang xue nuo | 60.2 mg/100g (anthocyanin^$^) | Boseokheukchal; 58.7 mg/100g (anthocyanin^$^) |
| JJ603Black | 2018 | N.A. | 58 mg/100g (anthocyanin^$^) | Heuknam; 51 mg/100g (anthocyanin^$^) |

^#^: The breeding pedigree of Heukjinmi contains Hongjinju, a red rice cultivar, as one of parents.

*: Not available.

^: Each unit for anthocyanin (or cyanidin 3-glucoside) contents in seeds was obtained from http://www.nics.go.kr/api/breed.do?m=100000128&homepageSeCode=nics without any changes.

^$^: The exact meaning of anthocyanin was not available from the database of the National Institute of Crop Science (http://www.nics.go.kr/api/breed.do?m=100000128&homepageSeCode=nics).
