# Supplementary material for: Reinterpretation of anthocyanins biosynthesis in developing black rice seeds through gene expression analysis
Source: PLoS One. 2023 Jun 2;18(6):e0286539. doi: 10.1371/journal.pone.0286539 (PMC10237452; doi:10.1371/journal.pone.0286539)
Supplement: S6 Table — (DOCX) [file pone.0286539.s010.docx]

**S6 Table. The correlation coefficient, in Dongjin (white rice), Geonganghongmi (red rice), Jeokjinju(red rice), Boseokheukchal (black rice), Heukjinju, Heukjinmi, and Heukseol (black rice), obtained from correlation analysis between the quantity of cyanidin 3-glucoside in hulled rice seeds and the expression levels of each gene involved in the biosynthetic pathway of anthocyanins in seeds at twenty days after heading and between the expression levels of these genes.**

| Variables | C3G^#^ | *bHLH1* | *bHLH2* | *MYB* | *WD40* | *PAL* | *C4H* | *4CL* | *HCT* | *CCR* | *CAD* | *CHS* | *CHI* | *F3H* | *F3´H* | *DFR* | *ANS* | *LAR* |
| --- | --- | --- | --- | --- | --- | --- | --- | --- | --- | --- | --- | --- | --- | --- | --- | --- | --- | --- |
| C3G | - | 0.6886^¶^  0.0006^^^ | 0.5507  0.0097 | 0.7815  <0.0001 | 0.7176  0.0003 | 0.2371  0.3008 | 0.3984  0.0736 | -0.4291  0.0523 | -0.2545  0.2656 | -0.5085  0.0186 | -0.3804  0.0889 | 0.8532  <0.0001 | 0.4317  0.0507 | 0.7797  <0.0001 | 0.9259  <0.0001 | 0.7929  <0.0001 | 0.7341  0.0002 | -0.3753  0.0936 |
| *bHLH1* | - | - | 0.8485  <0.0001 | 0.8715  <0.0001 | 0.9636  <0.0001 | 0.7821  <0.0001 | 0.4680  0.0324 | -0.0182  0.9376 | 0.0600  0.7961 | -0.2105  0.3598 | 0.1245  0.5908 | 0.9046  <0.0001 | 0.9036  <0.0001 | 0.8913  <0.0001 | 0.8212  <0.0001 | 0.9577  <0.0001 | 0.9264  <0.0001 | -0.0328  0.8879 |
| *bHLH2* | - | - | - | 0.6499  0.0014 | 0.7443  0.0001 | 0.6212  0.0027 | 0.1798  0.4354 | -0.2308  0.3142 | 0.0014  0.9952 | -0.1861  0.4192 | -0.0215  0.9263 | 0.8720  <0.0001 | 0.7828  <0.0001 | 0.8765  <0.0001 | 0.5567  0.0088 | 0.8624  <0.0001 | 0.6559  0.0012 | 0.2682  0.2398 |
| *MYB* | - | - | - | - | 0.8582  <0.0001 | 0.7357  0.0001 | 0.5141  0.0171 | -0.1118  0.6294 | -0.0273  0.9066 | -0.2112  0.3581 | -0.1606  0.4869 | 0.7949  <0.0001 | 0.7054  0.0004 | 0.7552  <0.0001 | 0.8397  <0.0001 | 0.9281  <0.0001 | 0.9578  <0.0001 | -0.1941  0.3993 |
| *WD40* | - | - | - | - | - | 0.7210  0.0002 | 0.6108  0.0033 | 0.0968  0.6762 | 0.1497  0.5173 | -0.1473  0.5239 | 0.1413  0.5411 | 0.8865  <0.0001 | 0.8765  <0.0001 | 0.9023  <0.0001 | 0.8832  <0.0001 | 0.9088  <0.0001 | 0.9013  <0.0001 | -0.0680  0.1902 |
| *PAL* | - | - | - | - | - | - | 0.5196  0.0158 | 0.3068  0.1762 | 0.3755  0.0935 | 0.2711  0.2346 | 0.3006  0.1855 | 0.5126  0.0175 | 0.8608  <0.0001 | 0.5444  0.0107 | 0.4321  0.0505 | 0.7376  0.0001 | 0.7744  <0.0001 | 0.2976  0.1902 |
| *C4H* | - | - | - | - | - | - | - | 0.3257  0.1497 | 0.4449  0.0433 | 0.2645  0.2467 | 0.2228  0.3317 | 0.4069  0.0672 | 0.4997  0.0211 | 0.4803  0.0275 | 0.5769  0.0062 | 0.4570  0.0373 | 0.4970  0.0219 | 0.1468  0.5255 |
| *4CL* | - | - | - | - | - | - | - | - | 0.8207  <0.0001 | 0.7755  <0.0001 | 0.4804  0.0275 | -0.2868  0.2074 | 0.2953  0.1937 | -0.1502  0.5157 | -0.1339  0.5629 | -0.1937  0.4003 | -0.0513  0.8252 | 0.3616  0.1072 |
| *HCT* | - | - | - | - | - | - | - | - | - | 0.9134  <0.0001 | 0.2459  0.2826 | -0.1134  0.6247 | 0.3911  0.0796 | 0.0350  0.8804 | -0.0657  0.7773 | -0.0291  0.9005 | -0.0179  0.9388 | 0.6726  0.0008 |
| *CCR* | - | - | - | - | - | - | - | - | - | - | 0.2034  0.3764 | -0.3855  0.0844 | 0.1585  0.4926 | -0.2271  0.3222 | -0.3806  0.0887 | -0.2523  0.2698 | -0.2506  0.2732 | 0.7488  <0.0001 |
| *CAD* | - | - | - | - | - | - | - | - | - | - | - | -0.0987  0.6704 | 0.3296  0.1446 | -0.0182  0.9378 | -0.1340  0.5625 | -0.0683  0.7685 | -0.0004  0.9987 | 0.1780  0.4401 |
| *CHS* | - | - | - | - | - | - | - | - | - | - | - | - | 0.7441  0.0001 | 0.9732  <0.0001 | 0.8690  <0.0001 | 0.9345  <0.0001 | 0.7960  <0.0001 | -0.0602  0.7955 |
| *CHI* | - | - | - | - | - | - | - | - | - | - | - | - | - | 0.7949  <0.0001 | 0.6244  0.0025 | 0.8209  <0.0001 | 0.7702  <0.0001 | 0.2973  0.1906 |
| *F3H* | - | - | - | - | - | - | - | - | - | - | - | - | - | - | 0.8213  <0.0001 | 0.9058  <0.0001 | 0.7400  0.0001 | 0.0868  0.7082 |
| *F3´H* | - | - | - | - | - | - | - | - | - | - | - | - | - | - | - | 0.8344  <0.0001 | 0.8522  <0.0001 | -0.3475  0.1227 |
| *DFR* | - | - | - | - | - | - | - | - | - | - | - | - | - | - | - | - | 0.9244  <0.0001 | -0.0256  0.9124 |
| *ANS* | - | - | - | - | - | - | - | - | - | - | - | - | - | - | - | - | - | -0.2458  0.2828 |
| *LAR* | - | - | - | - | - | - | - | - | - | - | - | - | - | - | - | - | - | - |

^#^: C3G (cyanidin 3-glucoside), quantity of cyanidin 3-glucoside (μg/g); *bHLH*: basic helix-loop-helix gene; *MYB*: myb gene; *WD40*: tryptophan-aspartic acid repeat protein gene; *PAL*: *phenylalanine ammonia-lyase*; *C4H*: *cinnamate 4-hydroxylase*; *4CL*: *4-coumarate: CoA ligase*; *CCR*: *cinnamoyl-CoA reductase*; *CAD*: *cinnamyl alcohol dehydrogenase*; *CHS*: *chalcone synthase*; *CHI*: *chalcone isomerase*; *F3H*: *flavanone 3-hydroxylase*; *DFR*: *dihydroflavonol 4-reductase*; *F3´H*: *flavonoid 3´-hydroxylase*; *ANS*: *anthocyanidin synthase*; *LAR*: *leucoanthocyanidin reductase*. ^¶^: correlation coefficient. ^^^: *p* value.
